# Supplementary material for: The lncRNA GATA6-AS epigenetically regulates endothelial gene expression via interaction with LOXL2
Source: Nat Commun. 2018 Jan 16;9:237. doi: 10.1038/s41467-017-02431-1 (PMC5770451; doi:10.1038/s41467-017-02431-1)
Supplement: Supplementary file 1 — Supplementary Information [file 41467_2017_2431_MOESM1_ESM.pdf]

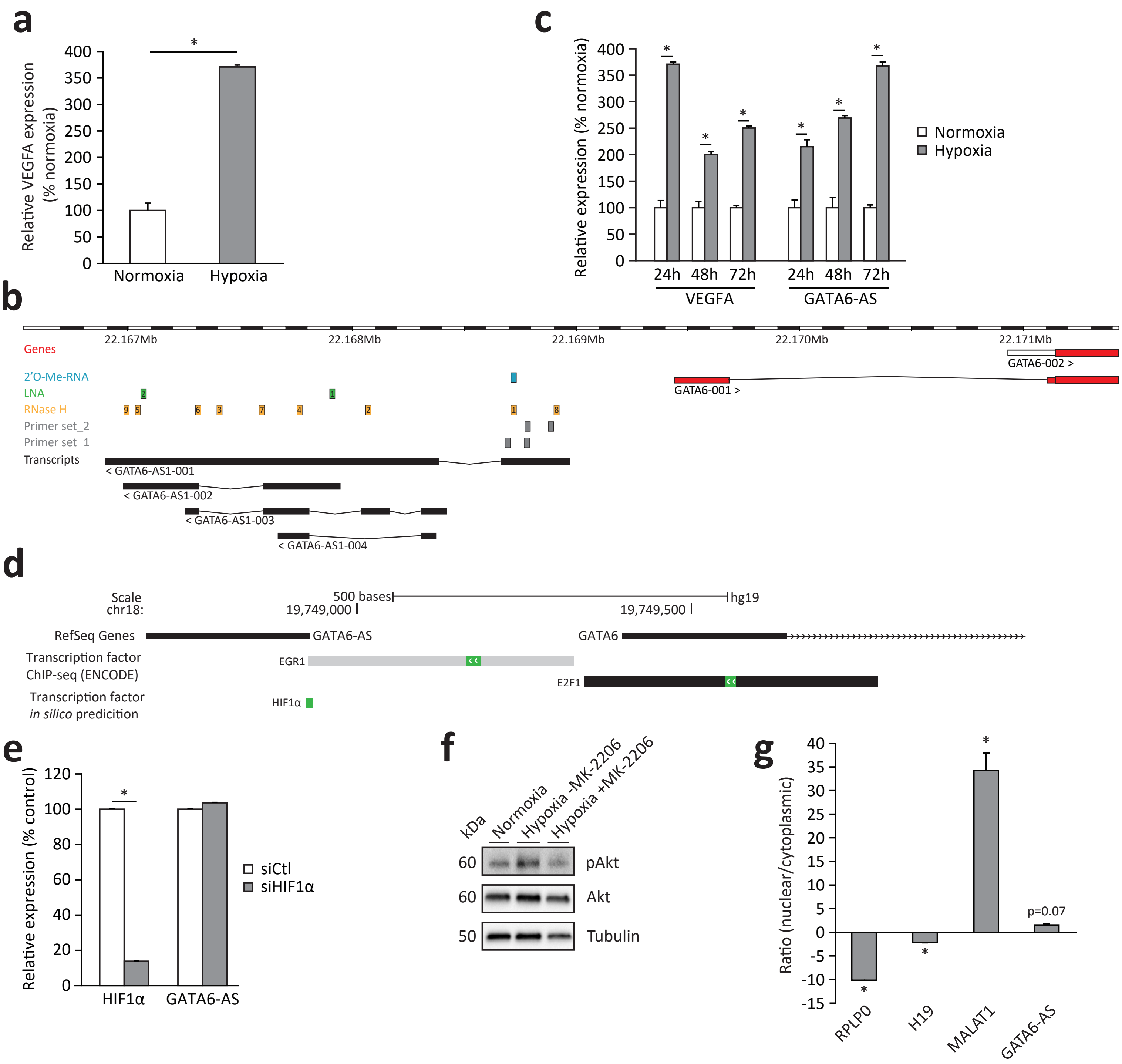

Supplementary Figure 1: The antisense transcript of GATA6 is up-regulated by hypoxia in an Akt-dependent manner. a HUVECs were exposed to hypoxia (24h, 0.2% O_2) or kept under normoxic conditions and induction of hypoxia was assayed by RT-qPCR using primers targeting VEGFA, normalized to RPLP0 mRNA (n=4; SEM; * t-test p<0.05). **b** Schematic overview of the human GATA6/GATA6-AS locus and oligonucleotide sequences used in this study. Transcripts (black) indicate the four annotated GATA6-AS transcripts and genes (red) GATA6. Primer pairs for RT-qPCR (grey; set 1 and 2), DNA oligonucleotides for RNase H cleavage (yellow; 1-9), LNA GapmeRs (green; 1, 2), and the 2'O-Me-RNA probe, used for RNA affinity selection (blue) are indicated at their respective positions by colored boxes. Scales at the top refer to chromosome 18. **c** HUVECs were exposed to hypoxia (0.2% O_2) for 24h, 48h and 72h or kept under normoxic conditions and GATA6-AS expression was analyzed by RT-qPCR. Relative expression levels were normalized to RPLP0 mRNA. VEGFA is shown as hypoxia-induced positive control (n=3-4; SEM; * t-test p<0.05). **d** Snapshot of the human GATA6/GATA6-AS locus with binding sites for E2F1 and EGR1. Downloaded and modified from UCSC genome browser (Homo sapiens: GRCh38/hg38). *In silico* analysis of transcription factor binding was performed using Promo software (http://bit.ly/1LkHwon). **e** HUVECs were transfected with siRNAs targeting HIF1α or with control siRNAs and exposed to hypoxia (24h, 0.2% O_2). Subsequently, RNA was isolated, DNase digested and relative expression levels of HIF1α and GATA6-AS were analyzed by RT-qPCR and normalized to RPLP0 mRNA levels (n=3; SEM; * t-test p<0.05). **f** Akt phosphorylation was inhibited in HUVECs by addition of MK-2206 to the culture medium 1h prior to hypoxic treatment (24h, 0.2% O_2). As controls, cells were incubated under normoxic conditions. Akt and phosphorylated Akt (pAkt), which represents the activated form and is known to be increased under hypoxia, were measured by western blotting. Tubulin was used as loading control (n=2; representative images are shown). **g** RNA from hypoxic (24h, 0.2% O_2) HUVECs was isolated from nuclear and cytoplasmic fractions and analyzed by RT-qPCR using primers targeting the indicated transcripts (n=4; SEM; * t-test p<0.05).

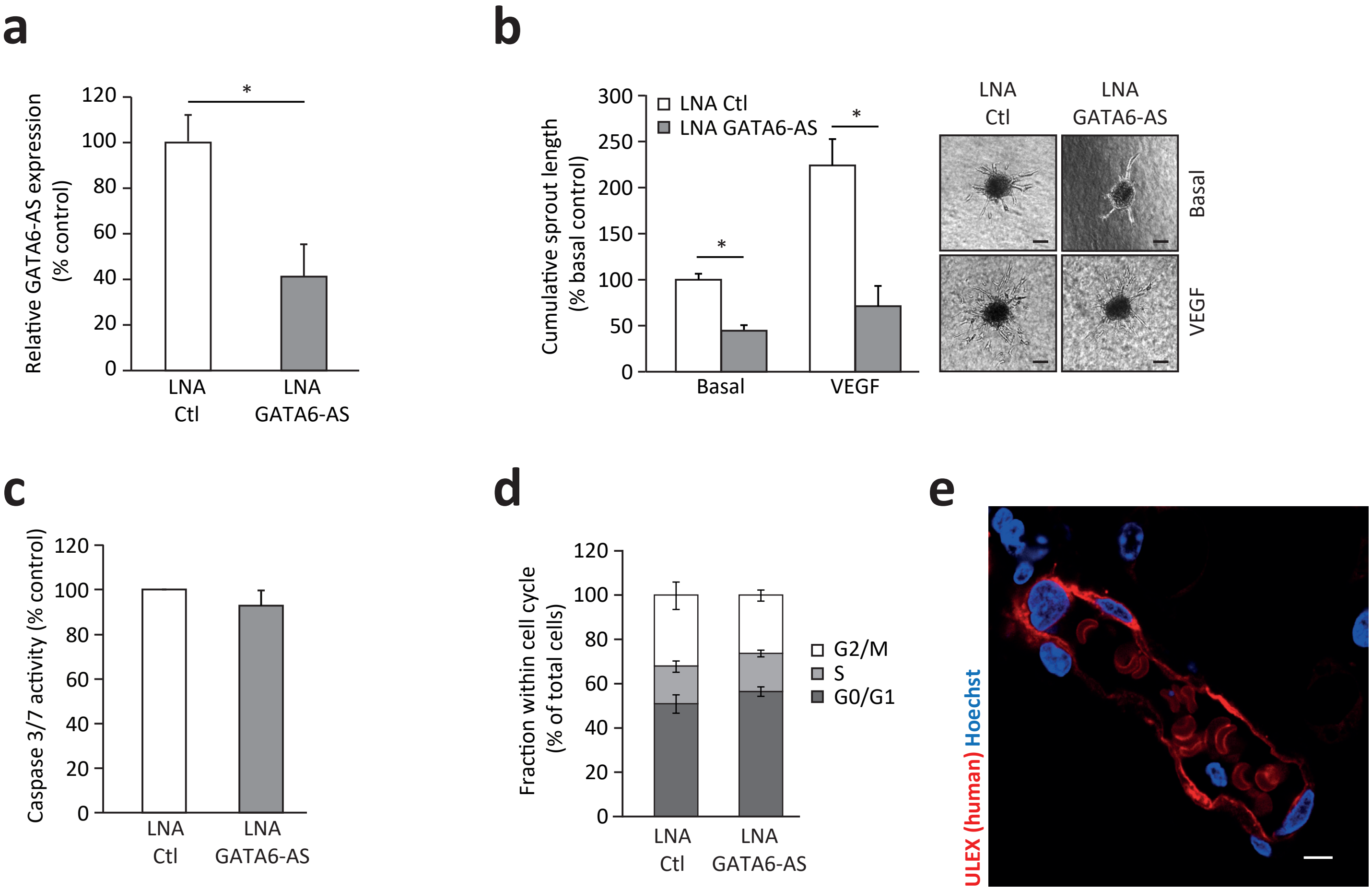

Supplementary Figure 2: GATA6-AS regulates endothelial phenotype and function. a HUVECs were transfected with an alternative GapmeR (#2, see Supplementary Fig. 1b) targeting GATA6-AS or with control GapmeRs and relative expression of GATA6-AS was determined by RT-qPCR, normalized to RPLP0 mRNA (n=6; SEM; * t-test p<0.05). **b** GATA6-AS-silenced cells (alternative GapmeR #2) or control cells were used for *in vitro* spheroid sprouting assays under basal conditions and VEGFA (50ng/ml) stimulation (n=5-6; SEM; * t-test p<0.05; representative images are shown; scale bars are 50µm). **c** HUVECs were transfected with GapmeRs targeting GATA6-AS or with control GapmeRs and caspase 3/7 activity was determined after 48h by luminescence measurements (n=11; SEM). **d** HUVECs were transfected with GapmeRs targeting GATA6-AS or with control GapmeRs and subjected to cell cycle analysis using BrdU-assays (n=4; SEM). **e** Matrigel basement matrix plugs containing spheroids derived from GATA6-AS or control GapmeR treated HUVECs were injected subcutaneously in immunodeficient mice. Plugs were harvested 21 days later and used for immunohistochemistry detecting human endothelial cells (ulex rhodamine, red). Nuclei were stained with Hoechst (blue) and erythrocytes were detected based on their autofluorescence (a representative image of a perfused vessel originating from HUVECs is shown; scale bar is 5µm).

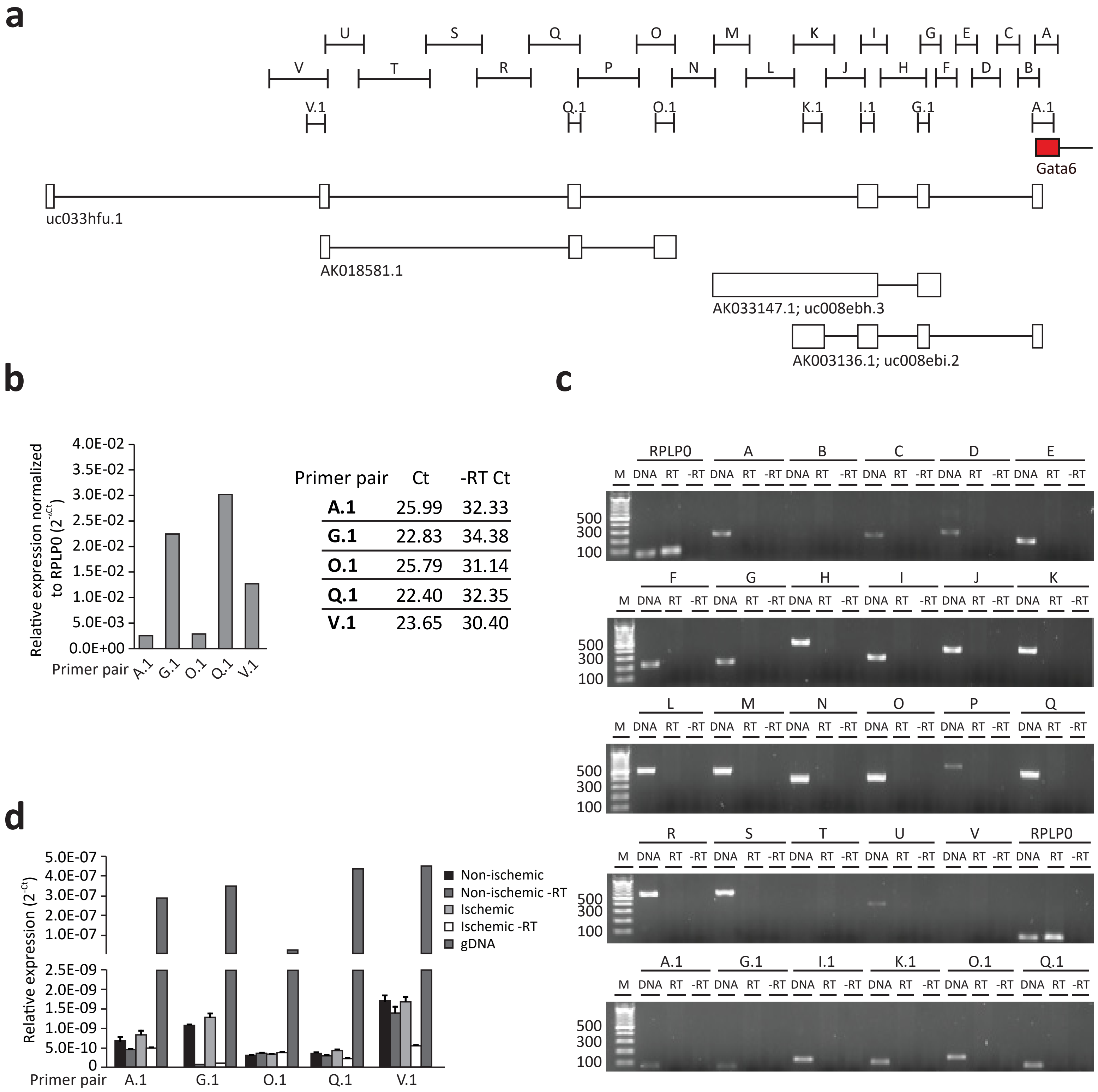

Primer pair	Ct	-RT Ct
**A.1**	25.99	32.33
**G.1**	22.83	34.38
**O.1**	25.79	31.14
**Q.1**	22.40	32.35
**V.1**	23.65	30.40

Supplementary Figure 3: Embryonal transcripts from the mouse Gata6-AS locus are absent in mouse endothelial H5V cells and in muscle tissue. a Schematic view of the mouse Gata6/Gata6-AS locus on chromosome 18 (GRCm38/mm10). The first exon of Gata6 is shown in red, putative Gata6-AS transcripts in white. Primer pairs (A-Q.1) used to cover the locus by RT-PCR are shown at the top. **b** Expression of putative Gata6-AS transcripts was assayed by RT-qPCR using different primer pairs (A.1-V.1). RNA was derived from embryonic hearts (Swiss mice; E12.5) and data was normalized to RPLP0 mRNA. Left: Relative expression levels. Right: $2^{-\Delta Ct}$, Ct and -RT Ct values (*n*=1). **c** Expression of putative mouse Gata6-AS transcripts was assayed by semiquantitative RT-PCR in mouse endothelial H5V cells. DNase-digested total RNA was reverse transcribed and subjected to PCR using the indicated primer pairs. RPLP0 served as positive control for RT reaction, genomic DNA as controls for primer functionality and -RT reactions as negative controls (*n*=1). **d** Expression of putative Gata6-AS transcripts was assayed by RT-qPCR in non-ischemic and ischemic muscle from adult mice 21 days after induction of ischemia, using the indicated primer pairs (*n*=3 mice per condition; SEM).

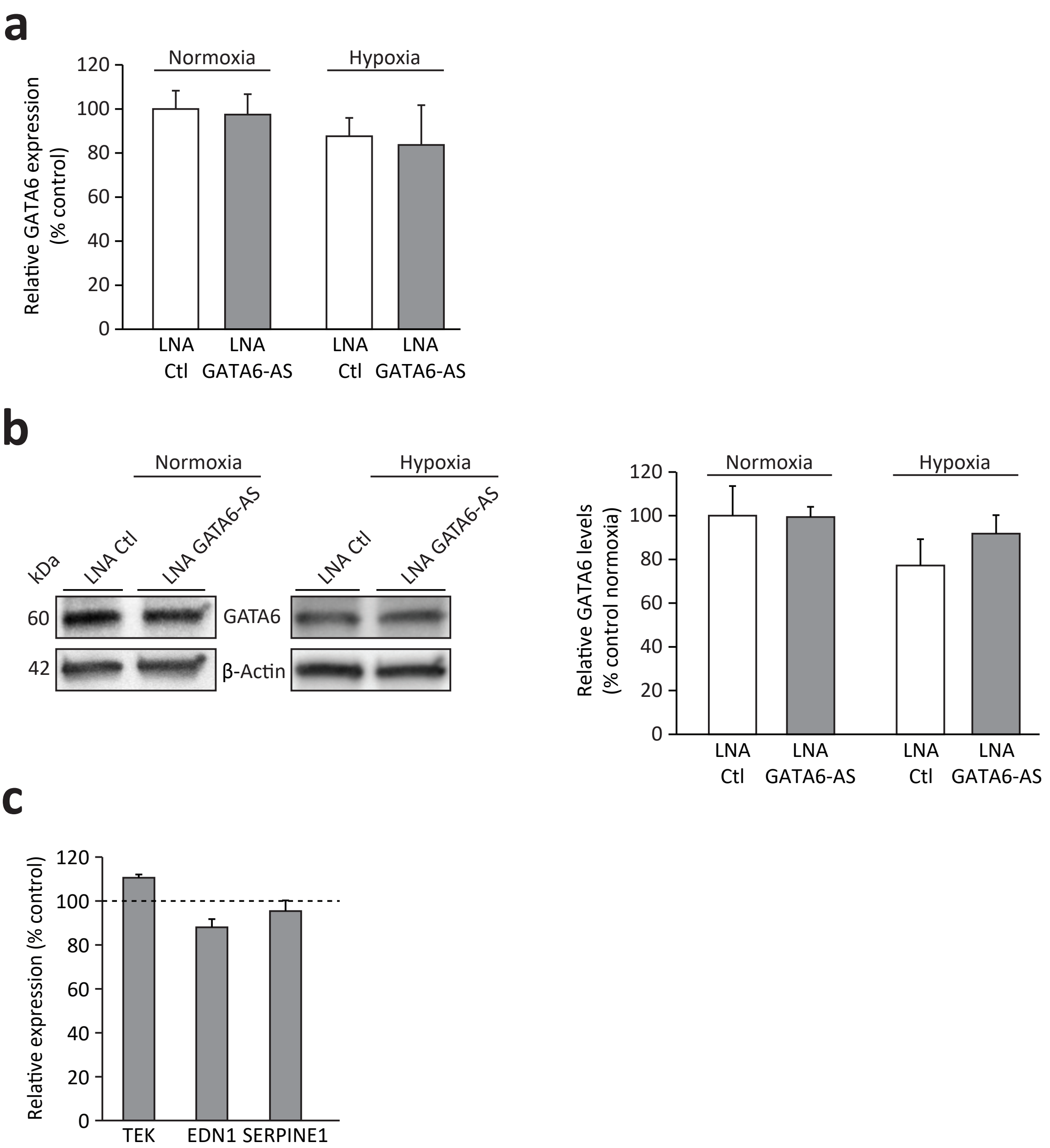

Supplementary Figure 4: GATA6-AS has no *cis*-regulatory function. a, b, c GATA6-AS expression was silenced in HUVECs by GapmeR transfection. **a** HUVECs were exposed to hypoxic (0.2% O_2) or normoxic control conditions for 24h. The relative expression of GATA6 mRNA was determined by RT-qPCR and expression values were normalized to RPLP0 mRNA (*n*=3; SEM). **b** HUVECs were exposed to hypoxic (0.2% O_2) or normoxic control conditions for 24h. Left: GATA6 protein levels were assayed by western blotting (representative images are shown). Right: Quantification of GATA6 protein levels, normalized to β-Actin (*n*=6; SEM). **c** Relative expression of the GATA6 target genes TEK, EDN1, and SERPINE1 was determined by RT-qPCR. Expression levels were normalized to RPLP0 mRNA (*n*=3; SEM).

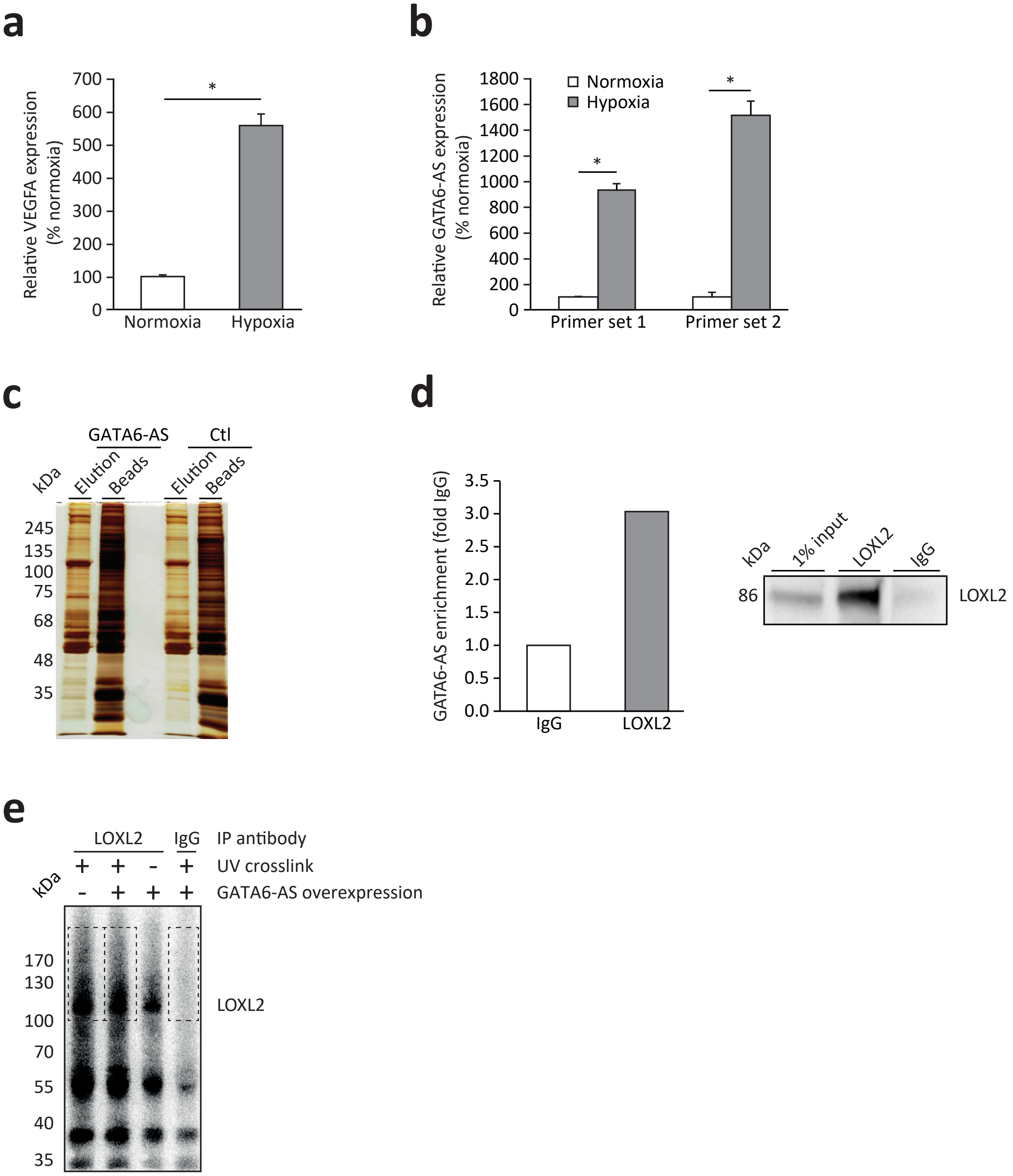

Supplementary Figure 5: GATA6-AS is hypoxia-induced and interacts with LOXL2. **a** Hypoxic conditions (24h, 0.2% O_2) in HeLa cells were assayed by RT-qPCR using primers targeting VEGFA. Expression was normalized to RPLP0 mRNA (*n*=4; SEM; * *t*-test *p*<0.05). **b** Regulation of GATA6-AS in HeLa cells by hypoxia (24h, 0.2% O_2) was confirmed by RT-qPCR, using two primer sets targeting GATA6-AS. Values were normalized to RPLP0 mRNA (*n*=4; SEM; * *t*-test *p*<0.05). **c** Eluates and beads fractions of endogenous GATA6-AS-protein-complexes isolated from HeLa cell lysate by 2'O-Me-RNA affinity selection were analyzed by SDS PAGE and silver staining. As control, scrambled 2'O-Me-RNA probes were used for affinity selection (*n*=7; a representative image is shown). **d** HUVECs were exposed to hypoxia (24h, 0.2% O_2) and UV_{254}-irradiated. Cell lysate was used for RNA immunoprecipitation using LOXL2 antibodies or IgG isotype controls. Left: Co-purified RNA was assayed for GATA6-AS enrichment by RT-qPCR. Right: IP specificity was controlled by LOXL2 western blotting (*n*=1). **e** Autoradiography of LOXL2-iCLIP membrane with bound ^{32}P-labeled RNA-protein-complexes comparing UV-irradiation, GATA6-AS overexpression, LOXL2 and isotype control immunoprecipitated material from HUVEC cell lysate. Boxed regions were cut out and subjected to RNA isolation and library preparation (*n*=1).

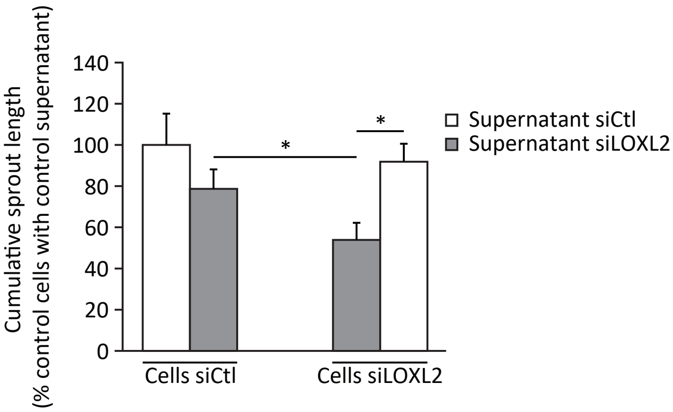

Supplementary Figure 6: LOXL2 sprouting phenotype is driven by an extracellular depletion of LOXL2. LOXL2-silenced cells or control cells were used for *in vitro* spheroid sprouting assays in the presence of supernatants from control or siLOXL2 treated cells. Therefore, supernatant derived from control cells or LOXL2-silenced cells was collected, concentrated by ultracentrifugation and incubated with the respective spheroids (see labels below bar graph) during sprout formation (n=6; SEM; * t-test p<0.05).

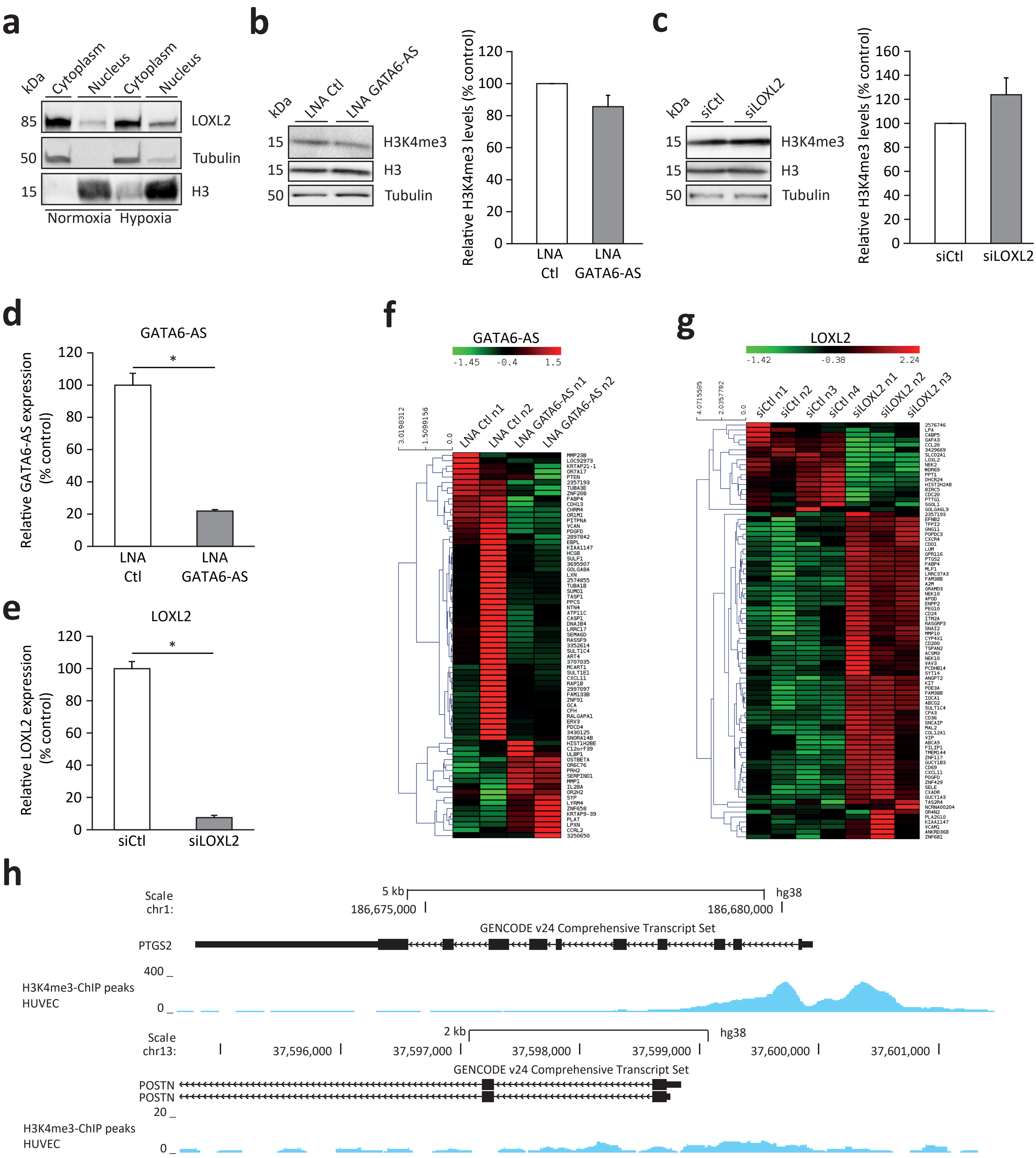

Supplementary Figure 7: GATA6-AS acts as negative regulator of nuclear LOXL2 function in endothelial cells. a HUVECs were exposed to hypoxia (0.2% O_2) or kept under normoxic conditions and LOXL2 levels of cytoplasmic and nuclear fractions were analyzed by western blotting. Tubulin and H3 were used as cytoplasmic and nuclear markers, respectively (*n*=3; representative images are shown). **b, c** HUVECs were transfected with GapmeRs or siRNAs targeting GATA6-AS or LOXL2, respectively and exposed to hypoxia (24h, 0.2% O_2). Left: H3K4me3, H3 as well as tubulin levels were assayed by western blotting (representative images are shown). Right: For quantification, H3K4me3 levels were normalized to tubulin (*n*=3-6; SEM). **d, e** HUVECs were transfected with GapmeRs or siRNAs targeting GATA6-AS or LOXL2, respectively and relative expression of GATA6-AS or LOXL2 was determined by RT-qPCR. Expression levels were normalized to RPLP0 mRNA (*n*=3-6; SEM; * *t*-test $p<0.05$). **f, g** Gene expression profiling of GATA6-AS-silenced and LOXL2-silenced HUVECs (*n*=2-4). **h** Snapshot of the human PTGS2 and POSTN locus with H3K4me3 ChIP-sequencing peaks, downloaded and modified from UCSC genome browser (Homo sapiens: GRCh38/hg38).

Figure 4a

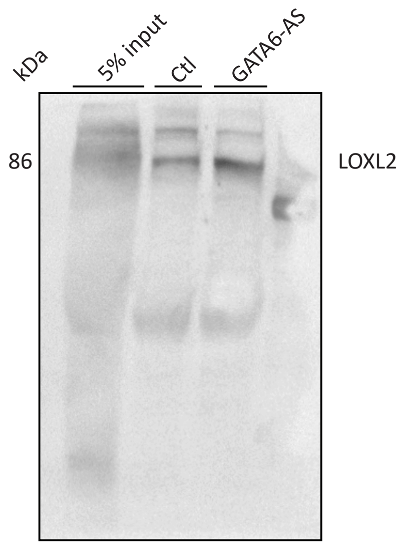

Figure 5d

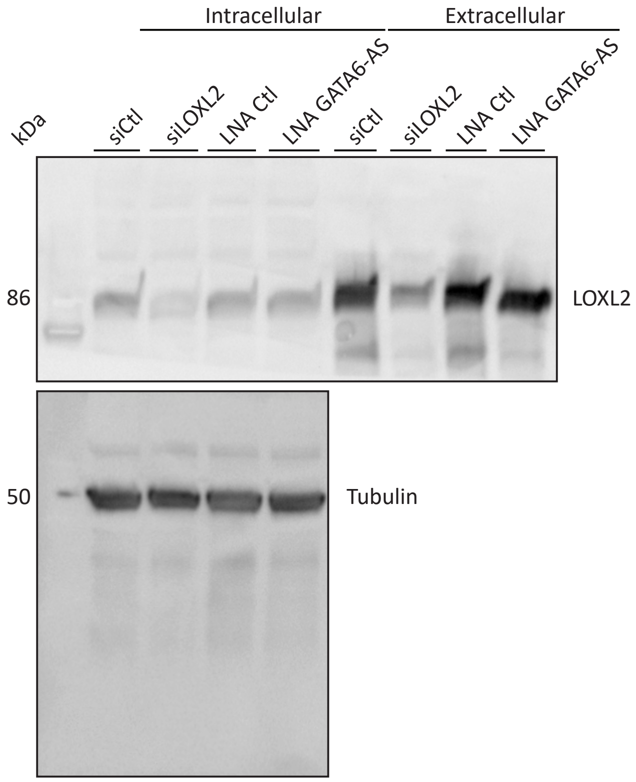

Figure 6a

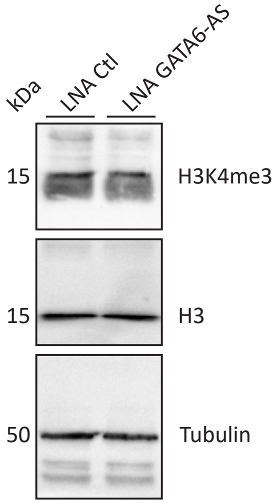

Figure 6b

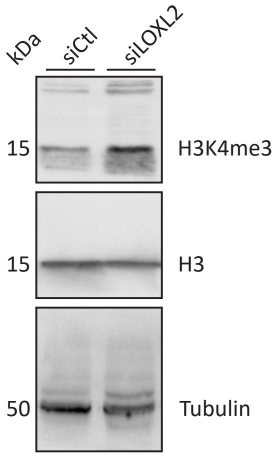

Supplementary Figure 8: Western blot images used to compose result panels in indicated main figures.

Supplementary Figure 1f

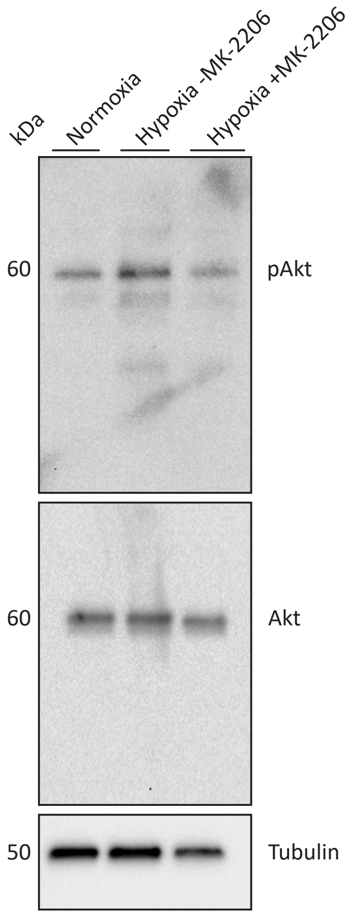

Supplementary Figure 7a

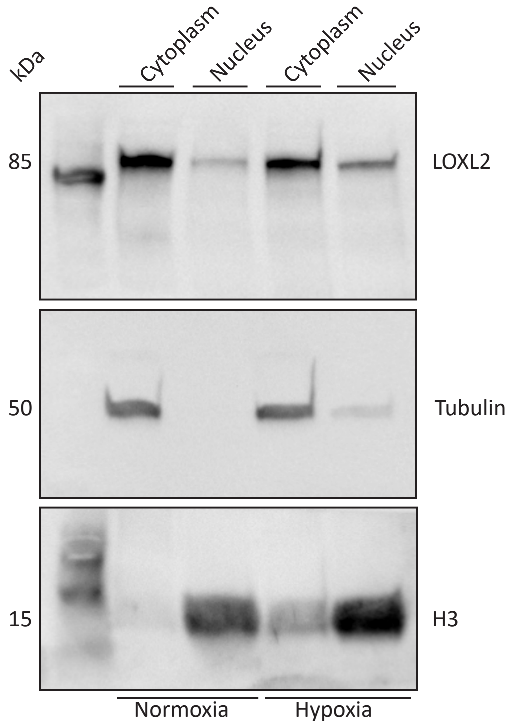

Supplementary Figure 7b

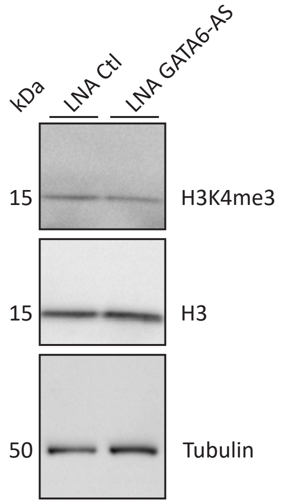

Supplementary Figure 7c

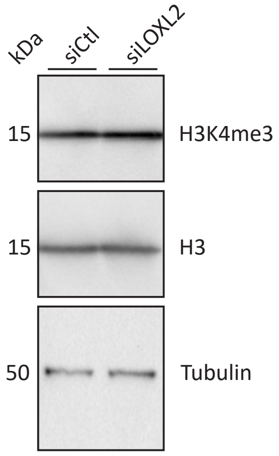

Supplementary Figure 9: Western blot images used to compose result panels in indicated supplementary figures.

Supplementary Table 1: Hypoxia-regulated lncRNAs in endothelial cells. HUVECs were cultured under hypoxic (24h, 0.2% O_2) or normoxic conditions and gene expression changes were assayed by deep sequencing of ribo-minus RNA. Significantly regulated lncRNAs are shown in descending order.

		FPKM (log_2)					
		Normoxia 24h		Hypoxia 24h			
Short name	Locus (GRCh37/hg19)	n1	n2	n1	n2	Ratio norm/hyp (log_2)	p value
H19,MIR675	11:2016405-2022700	0.77	0.56	4.32	8.41	3.26	0.0001
MIR210HG	11:565659-568457	2.02	2.26	16.06	17.55	2.97	0.0001
RP11-923I11.1	12:52203488-52206648	0.56	0.33	2.97	2.75	2.69	0.0001
**GATA6-AS / RP11-627G18.3**	**18:19746858-19748929**	**0.42**	**0.40**	**2.10**	**3.03**	**2.64**	**0.0002**
XIST	X:73040485-73072588	0.61	2.83	2.18	18.70	2.60	0.0001
AC004383.4,MIR424	X:133677366-133680741	8.77	6.62	55.22	38.13	2.60	0.0001
AC009236.1	2:45392008-45550434	0.23	0.56	0.30	4.38	2.55	0.0010
AC009404.2	2:118591512-118599234	0.31	0.37	1.51	1.87	2.33	0.0010
LINC00342	2:96472865-96481963	0.55	0.42	1.69	2.86	2.24	0.0001
AC083843.1	8:135804262-135810515	0.27	0.18	0.94	1.18	2.24	0.0001
RP11-379B18.5	3:125546080-125629115	0.23	0.28	1.00	1.27	2.18	0.0018
MEG3	14:101245746-101327368	12.84	15.27	54.79	69.62	2.15	0.0001
MALAT1	11:65265232-65273940	211.71	169.01	570.41	942.98	1.99	0.0001
NEAT1	11:65190244-65213011	16.57	10.15	33.09	70.10	1.95	0.0001
RP11-48B3.4	8:81453534-81455339	0.37	0.43	1.30	1.69	1.91	0.0001
RP1-67M12.2	6:22135185-22147422	0.21	0.32	0.76	1.16	1.86	0.0014
AC012065.7	2:20877568-20879005	3.19	2.43	11.07	9.06	1.84	0.0001
MEG9	14:101536247-101539274	0.68	0.30	1.21	2.21	1.80	0.0027
RP11-48B3.3	8:81447240-81451370	0.25	0.30	0.86	1.05	1.79	0.0002
RP11-9G1.3	4:133996465-134070271	1.25	1.13	4.42	3.81	1.79	0.0016
RP11-553L6.5	3:114033347-114035026	0.53	0.69	1.84	2.22	1.74	0.0001
RP11-354K1.1	1:211809247-211827923	0.86	0.45	2.35	2.04	1.74	0.0011
RP5-1057J7.6	1:23607801-23613245	0.62	0.65	1.58	2.54	1.69	0.0001
RP11-222A11.1	10:67330207-67526299	0.94	0.45	1.27	3.15	1.68	0.0001
LINC00115	1:761585-762902	0.56	0.18	1.00	1.33	1.66	0.0049
RP11-31E23.1	1:198776621-198906558	1.15	2.06	2.82	7.08	1.62	0.0005
RP5-1070A16.1	1:98676302-98738214	0.40	0.37	0.79	1.61	1.62	0.0006
RP11-361F15.2	6:147708799-147711601	0.33	0.46	1.13	1.20	1.56	0.0043
AC156455.1	12:122501211-122506522	2.87	3.49	8.47	9.05	1.46	0.0005
ZNF518A	10:97889471-98031333	2.58	2.56	5.34	8.42	1.42	0.0001
AF127936.7	21:16195016-16254296	1.84	1.25	4.43	3.71	1.40	0.0020
CTC-428G20.3	5:114539712-114541943	0.55	0.36	0.85	1.51	1.38	0.0018
RP11-158H5.7,ZSCAN30	18:32820993-32870196	4.11	4.94	10.15	12.72	1.34	0.0001
RP11-37B2.1	8:90621628-90769955	2.25	1.42	4.41	4.73	1.32	0.0011
SH3RF3-AS1	2:109743782-109745386	0.97	1.19	2.28	3.07	1.31	0.0008
AC007204.2,ZNF93	19:20011721-20074273	1.52	2.31	2.79	6.46	1.27	0.0031
RP11-373L24.1	2:61153043-61155287	0.90	0.61	1.50	2.10	1.25	0.0009
CTC-444N24.8	19:57772721-57774106	1.06	1.54	2.68	3.48	1.24	0.0009
MIR4720	16:81416873-81424489	1.63	1.66	4.11	3.47	1.21	0.0004
CTC-444N24.11	19:57815672-57819930	3.52	3.82	7.88	8.67	1.17	0.0001
RP11-121C2.2	4:47842138-47846356	0.33	0.30	0.49	0.90	1.14	0.0040
FTX	X:73467698-73513362	9.16	8.54	17.62	21.00	1.13	0.0006
HCG11	6:26522075-26526807	1.43	1.45	3.14	3.11	1.12	0.0001
MIRLET7DHG	9:96938883-96966818	2.52	2.27	4.11	6.00	1.08	0.0007
RP11-890B15.3	11:130736148-130740142	0.67	0.81	1.26	1.83	1.06	0.0009
LINC00472	6:72054046-72130472	1.73	2.60	3.31	5.21	0.98	0.0048
RP3-523C21.1	6:132453054-132490514	72.06	57.09	134.26	117.73	0.96	0.0001
LINC00657	20:34633543-34638882	64.36	64.90	126.05	125.58	0.96	0.0001
RP11-220I1.1	9:37079892-37090398	9.14	8.51	15.60	18.53	0.95	0.0016
AC017048.3	2:177494567-177520686	10.48	8.71	19.90	16.79	0.93	0.0001
TUG1	22:31366662-31374831	18.59	17.16	28.42	33.62	0.80	0.0016
RP11-252A24.7	16:74481324-74483790	9.03	9.25	14.26	16.31	0.74	0.0012
RP1-140K8.5	6:3905143-3912213	4.17	3.80	6.77	6.41	0.73	0.0005
RP11-18F14.2	16:80631802-80636416	6.58	7.66	11.17	11.30	0.66	0.0020
RP11-159D12.2,RP11-159D12.5,SRSF1	17:56066398-56084707	95.81	102.37	59.76	64.97	-0.67	0.0016
RP11-327L3.1	9:35909479-35911683	4.61	6.98	2.12	3.95	-0.93	0.0020
CTD-2292M16.8,TTC5	14:20724716-20774970	12.94	19.74	7.24	9.45	-0.97	0.0020
DANCR	4:53578596-53586998	66.32	54.72	33.25	26.74	-1.01	0.0001
RP11-1094M14.11	17:33895138-33901811	3.67	3.58	1.40	1.87	-1.15	0.0014
LINC00116	2:110969105-111043433	46.38	45.35	23.75	17.23	-1.16	0.0001
RP11-253E3.3	12:3150602-3154116	2.17	2.17	0.89	0.93	-1.25	0.0003
RP11-160E2.6	17:19015312-19015949	26.46	21.18	8.61	9.76	-1.37	0.0001
SNORD3B-2	17:18966761-18967449	23.08	30.27	7.66	11.54	-1.47	0.0001
RP11-184M15.1	4:129489126-129491686	3.47	4.81	0.47	1.03	-2.46	0.0001

Supplementary Table 2: *In silico* analysis of GATA6-AS coding potential. All annotated splice variants of GATA6-AS were analyzed regarding their protein coding probabilities using the coding potential assessment tool CPAT (http://rna-cpat.sourceforge.net/). Analysis was done using default settings for human sequences and coding probabilities below 0.364 were considered non-coding.

Transcript variant	RNA size	ORF size	Ficket score	Hexamer score	Coding probability	Coding label
GATA6-AS1-001	1788	249	0.602	0.109	0.052	no
GATA6-AS1-002	669	279	0.696	0.034	0.062	no
GATA6-AS1-003	481	153	0.770	0.189	0.057	no
GATA6-AS1-004	194	48	0.533	-0.297	0.000	no

Supplementary Table 3: Proteins identified by mass spectrometry in GATA6-AS affinity selections. Endogenous GATA6-AS-protein-complexes were targeted by RNA affinity selection using 2’O-Me-RNA oligos and co-purified proteins were analyzed by mass spectrometry. As controls, scrambled oligos were used and proteins with unique peptide values ≥ 8 were considered for further evaluation. Log_2 fold changes over control selections were calculated ($LFQ_{GATA6\text{-}AS}$ - LFQ_{Ctl}), comparing total enrichment and enrichment in UV_{254}-irradiated samples and values were shaded largest (red) to smallest (orange). Identified proteins were analyzed for the indicated GO-term assignments and highlighted in yellow when applicable. Data are *n*=7.

Log_2 fold change (total)	Log_2 fold change (crosslink only)	Gene names	Protein names	Unique peptides	Nucleic acid binding	Response to hypoxia	Sprouting angiogenesis	Histone modification
68.46	44.33	LOXL2	Lysyl oxidase homolog 2	20				
64.66	48.92	MAGEB2	Melanoma-associated antigen B2	9				
63.25	27.32	CTSB	Cathepsin B;Cathepsin B light chain;Cathepsin B heavy chain	10				
61.81	23.76	TBRG4	Protein TBRG4	17				
57.44	22.04	KPNA2	Importin subunit alpha-1	16				
51.47	25.49	COPB2	Coatomer subunit beta	38				
50.38	27.61	FLNA	Filamin-A	67				
50.33	23.45	ARCN1	Coatomer subunit delta	30				
49.32	24.71	FLNC	Filamin-C	79				
40.66	28.70	PTBP1	Polypyrimidine tract-binding protein 1	18				
38.58	23.71	POP1	Ribonucleases P/MRP protein subunit POP1	21				
38.40	25.72	CLTC	Clathrin heavy chain 1	35				
38.24	27.12	GRSF1	G-rich sequence factor 1	11				
38.09	24.69	BRIX1	Ribosome biogenesis protein BRX1 homolog	19				
37.37	24.78	SEC22B	Vesicle-trafficking protein SEC22b	10				
32.42	2.97	CCT2	T-complex protein 1 subunit beta	33				
29.41	3.30	POLR3B	DNA-directed RNA polymerase III subunit RPC2	24				
28.74	26.46	TALDO1	Transaldolase	11				
26.48	23.94	POLR2E	DNA-directed RNA polymerases I, II, and III subunit RPABC1	10				
25.49	23.95	RAN	GTP-binding nuclear protein Ran	9				
25.39	1.27	VPS35	Vacuolar protein sorting-associated protein 35	25				
24.75	23.54	AK4	Adenylate kinase 4, mitochondrial	10				
22.95	21.13	PSMD1	26S proteasome non-ATPase regulatory subunit 1	46				
19.11	4.80	ILF2	Interleukin enhancer-binding factor 2	17				
17.63	3.77	MYBBP1A	Myb-binding protein 1A	38				
17.31	4.71	ENO1	Alpha-enolase	19				
16.93	0.67	EIF6	Eukaryotic translation initiation factor 6	8				
16.72	2.58	ELAVL1	ELAV-like protein 1	11				
13.77	0.63	PDHA1	Pyruvate dehydrogenase E1 component subunit alpha	9				
9.20	2.35	PWP1	Periodic tryptophan protein 1 homolog	20				
9.05	4.02	COLGALT1	Procollagen galactosyltransferase 1	23				
7.29	3.42	TFRC	Transferrin receptor protein 1	26				
6.35	2.65	RPL8	60S ribosomal protein L8	19				
6.35	0.70	CDC73	Parafibromin	28				
5.81	1.47	NPEPPS	Puromycin-sensitive aminopeptidase	42				
5.26	2.66	IDH1	Isocitrate dehydrogenase [NADP] cytoplasmic	21				
5.26	3.41	EEF2	Elongation factor 2	43				
5.13	0.23	CCT3	T-complex protein 1 subunit gamma	35				
4.75	2.23	RPL5	60S ribosomal protein L5	21				
4.38	2.25	GAPDH	Glyceraldehyde-3-phosphate dehydrogenase	18				
3.54	1.89	EIF4A1	Eukaryotic initiation factor 4A-I	22				
2.52	1.77	RPLP0;RPLP0P6	60S acidic ribosomal protein P0	20				
1.74	0.80	AIFM1	Apoptosis-inducing factor 1, mitochondrial	25				

Supplementary Table 4: Silencing of GATA6-AS diminishes the expression of hypoxia- and angiogenesis-related transcripts in endothelial cells. Genes found to be inversely regulated upon GATA6-AS and LOXL2 silencing were assayed for the GO-terms “positive regulation of angiogenesis” as well as “response to hypoxia”. Changes in expression levels upon GATA6-AS and LOXL2 silencing are shown.

		Fold Ctl	
**Description**	**Gene name**	**LNA GATA6-AS**	**siLOXL2**
Prostaglandin G/H synthase 2	PTGS2	-1.49	2.76
Rho-related GTP-binding protein RhoB	RHOB	-1.35	1.32
Neurofibromin	NF1	-1.33	1.09
Signal transducer CD24	CD24	-1.32	2.09
C-C motif chemokine 2	CCL2	-1.31	1.21
Intercellular adhesion molecule 1	ICAM1	-1.30	1.09
Homeobox protein Meis1	MEIS1	-1.29	1.29
Peptidyl-glycine alpha-amidating monooxygenase	PAM	-1.29	1.30
Serine/threonine-protein kinase D1	PRKD1	-1.28	1.42
Hematopoietic progenitor cell antigen CD34	CD34	-1.28	1.54
Interleukin-8	IL8	-1.28	1.15
Apoptotic protease-activating factor 1	APAF1	-1.27	1.41
Sushi repeat-containing protein SRPX2	SRPX2	-1.26	1.12
Vascular cell adhesion protein 1	VCAM1	-1.25	2.59
Periostin	POSTN	-1.23	1.43
Cellular tumor antigen p53	TP53	-1.23	1.07
Vascular endothelial growth factor receptor 1	FLT1	-1.22	1.44
TGF-beta receptor type-2	TGFBR2	-1.22	1.05
Baculoviral IAP repeat-containing protein 2	BIRC2	-1.21	1.12
Cysteinyl leukotriene receptor 2	CYSLTR2	-1.21	1.16
Platelet-derived growth factor subunit B	PDGFB	-1.21	1.06

Supplementary Table 5: Sequences of all oligonucleotides used in this study.

Name	Sequence (5'-3')
LNA GapmeRs	
LNA Ctl	AACACGTCTATACGC
LNA GATA6-AS	TCGGTAGCAATTTAA
LNA GATA6-AS_alternative	AAAGGAGCAATCACTT
siRNAs	
siCtl	CGUACGCGGAAUACUUCGA[dT][dT]
siLOXL2	CACAUAGGUGGUUCCUUCA[dT][dT]
siHIF1α	SASI_Hs02_00332063
Primers for RPLP0, GATA6-AS, H19, MALAT1	
RPLP0_f	TCGACAATGGCAGCATCTAC
RPLP0_r	ATCCGTCTCCACAGACAAGG
GATA6-AS_f_primer set 2	ATGCGCTTTTTGCCCTGAAG
GATA6-AS_r_primer set 2	AGGTCAGCTGGGGAATGTTG
GATA6-AS_f_primer set 1	ATTCCCCAGCTGACCTTTGG
GATA6-AS_r_primer set 1	CGGACACGACTGATGTGGAA
H19_f	TCAAGCCTGGGCCTTTGAAT
H19_r	GGCTGATGAGGTCTGGTTCC
MALAT1_f	GTGATGCGAGTTGTTCTCCG
MALAT1_r	CTGGCTGCCTCAATGCCTAC
Primers for VEGFA, HIF1α	
VEGFA_f	CCCTGATGAGATCGAGTACA
VEGFA_r	AGCAAGGCCCACAGGGATTT
HIF1α_f	GGCAGCAACGACACAGAAAC
HIF1α_r	TTTTCGTTGGGTGAGGGGAG
Antisense oligos for RNase H cleavage	
GATA6-AS_RNaseH#1	AAGCCCTTTTCCATTCTGCG
GATA6-AS_RNaseH#2	ACAACGCTTAGCTACGAGGT
GATA6-AS_RNaseH#3	CAGCCTGGACACAAACTGAG
GATA6-AS_RNaseH#4	ATTCCAGAGTTTTCTACCTT
GATA6-AS_RNaseH#5	CTGTGACCATGAAAAGGAGC
GATA6-AS_RNaseH#6	AGACATCCTTGTACTTGCAA
GATA6-AS_RNaseH#7	ACATTTGGTCGGAACTCACG
GATA6-AS_RNaseH#8	TAAGGTCGGTCTCACACAAC
GATA6-AS_RNaseH#9	CCTCGTTAGGTTGGAAAACCAG
Primers for RNase H cleavage	
GATA6-AS_RNaseH#1_f	CGGGTCGTCATGTACGGAAA
GATA6-AS_RNaseH#1_r	GTCCTCGGACACGACTGATG
GATA6-AS_RNaseH#2_f	GGTGGAGAGGTGCCTTGTAA
GATA6-AS_RNaseH#2_r	ACTCACAGTTACGTGCAGAGG
GATA6-AS_RNaseH#3_f	CGGAAATGGGTTGTGGGCAT
GATA6-AS_RNaseH#3_r	CACAGGACAGAGATCAGGCTC

GATA6-AS_RNaseH#4_f	GTGGCCGCATTTGGAAAAGG
GATA6-AS_RNaseH#4_r	TAAAGCCCCTGGATCCTGCT
GATA6-AS_RNaseH#5_f	ACCGATTCGACAACGTTCGG
GATA6-AS_RNaseH#5_r	CACTACCCATTCCCTCGTTAGG
GATA6-AS_RNaseH#6_f	GCCTGATCTCTGTCCTGTGT
GATA6-AS_RNaseH#6_r	AGCCCGAACGTTGTCGAATC
GATA6-AS_RNaseH#7_f	ACTCGGAATGCCTTGCTTTTG
GATA6-AS_RNaseH#7_r	ACCCATTTCCGTCAGTGCTTA
GATA6-AS_RNaseH#8_f	CCTAGCCCCTTCACAGCAAA
GATA6-AS_RNaseH#8_r	ATCCCGAGATAGGGTCGGAG
GATA6-AS_RNaseH#9_f	GAAGGGACCCGGCATTTTAC
GATA6-AS_RNaseH#9_r	CTGTCTGAATCAACTGTACGAG
2'O-Me-RNA probes	
GATA6-AS	mAmAmGmCmCmCmUmUmUmUmCmCmAmUmUmCmUm GmCmG/iSp9/rArCrGrArUrC/3deSBioTEG
Ctl	mGmCmAmAmGmGmAmAmCmGmUmGmUmGmAmGmA mCmUmA/iSp9/rArCrGrArUrC/3deSBioTEG
Primers for TEK, EDN1, SERPINE1, GATA6	
TEK_f	AGCCTGCTTCTGTGCTGTTC
TEK_r	CCACAGTTCCAGAAAGGAGCA
EDN1_f	TCCCACAAAGGCAACAGACC
EDN1_r	GTCTCCGACCTGGTTTGTCTT
SERPINE1_f	AGAGCGCTGTCAAGAAGACC
SERPINE1_r	CTGGAGACATCTGCATCCTGAA
GATA6_f	CACGAACAGGGCGATTTCCT
GATA6_r	TCTGTTGGGGGTAACGTCTG
Primers for GATA6-AS Northern blot probe	
GATA6-AS_f_NB	TAATACGACTCACTATAGGTATCTCTTCTGGGAGTCGCG
GATA6-AS_r_NB	TTTCAGCGTGGCCGCAT
Primers for truncated GATA6-AS	
GATA6-AS_f	AACTCTGGAATGGAGAGGCTG
GATA6-AS_r	TCTTCTGGGAGTCGCGCATT
iCLIP	
3’-RNA linker:	5’-Phosphate-UGAGAUCGGAAGAGCGGUUCAG-Puromycin-3’
LOXL2-IP+GATA6-AS overexpression	5’-Phosphate-NNGCCANNNAGATCGGAAGAGCGTCGTGgatcCTGAACCGC-3’
LOXL2-IP	5’-Phosphate-NNTCCGNNNAGATCGGAAGAGCGTCGTGgatcCTGAACCGC-3’
IgG-IP+GATA6-AS	5’-Phosphate-NNTGCCNNNAGATCGGAAGAGCGTCGTGgatcCTGAACCGC-3’

Primers for PTGS2, CYSTLR2, VCAM1, POSTN, PLAT, LOXL2	
PTGS2_f	TGAAACCCACTCCAAACACA
PTGS2_r	TGTGATCTGGATGTCAACACA
CYSTLR2_f	GCAAGGTATGGAGAGTTCCTC
CYSTLR2_r	TGCTGTCTCTGTACACCTGA
VCAM1_f	GACCACATCTACGCTGACAAT
VCAM1_r	GAGGGCCACTCAAATGAATCT
POSTN_f	GTTACAAGAAGAGGTCACCAAGG
POSTN_r	AACTTCCTCACGGGTGTGT
PLAT_f	TGTGGAGCAGTCTTCGTTTC
PLAT_r	TTCATCTCTGCAGATCACTTGG
LOXL2_f	CAGCGCTACTGGCCATTCT
LOXL2_r	TCTGGCTTGTACGCTTTCCG
Primers for ChIP	
ChIP_PTGS2_f	CGCTGCAAGAAGACGAAGAA
ChIP_PTGS2_r	TAAGTGCGATTGTACCCGGA
ChIP_POSTN_f	CCGACCCCTGATACGACTAT
ChIP_POSTN_r	GGACTAACTGCAACGGAGAG
ChIP_negative_control_f	GGATGCTTTTGTAAGCAAGTAACGA
ChIP_negative_control_r	GCTGGGTGCACCACATTAATAC
Primers for EndMT	
SM22_f	AAGAATGATGGGCACTACCG
SM22_r	ATGACATGCTTTCCCTCCTG
Calponin_f	CTGGCTGCAGCTTATTGATG
Calponin_r	CTGAGAGAGTGGATCGAGGG
Primers for mouse Gata6-AS locus	
mA_f	GAGCGATGTGCGAGAAGAAC
mA_r	GTAGAGAGCAGTTCCGACCC
mB_f	AAACAAACAGCGCTAGCCGA
mB_r	GTGGACTCGCTCCTACTCTG
mC_f	TCCTCAGAGTAGGAGCGAGT
mC_r	CGGGGTCTATGAAGATCCAGC
mD_f	GGAAACTTGGCTCGGAAGGA
mD_r	TTTTAAGAGCAGGGGCGAGG
mE_f	CTTTGGAATTCAACGCTCTGGG
mE_r	AAAGCTCAGAGTGAACGCGA
mF_f	TCGCGTTCACTCTGAGCTTT
mF_r	GATAACCCCGCCAGTGTTCA
mG_f	TGATACGGTTCTTAGCGGGC
mG_r	ATCCACTCCCACAAGAGTGT
mH_f	TTCGACAAGCTGGACATTGG
mH_r	ACGCAGTTAGGCAAGAACG
mI_f	CGCGGCCAAACACTTCTAAA
mI_r	TGGAAGCCGTGGTGGGAAT
mJ_f	TACCACAAGAGACTGGCACG
mJ_r	GGGGTTTGCTATTGTTCCAGG

mK_f	GAGCCTAGTTCCAGTCCTGT
mK_r	AGTCAACCGGACAGGTTTTATTT
mL_f	ACCTGTCCGGTTGACTAAGAC
mL_r	CTGCTGTGTTCCGGCTAGAT
mN_f	ACCAAACCACCACTTGCCTT
mN_r	GCGTGAGAATCGAACCCACA
mM_f	AGGATGTGGGTTCGATTCTC
mM_r	ATTAGCTCTGAAGCCTGAGG
mO_f	GTGTTGGTTCTCTTTCTGCCTG
mO_r	GGAGGGAAAATCTTTCGTCGG
mP_f	CCACCCAATGACCGACGAAA
mP_r	CCCCAAGGGGATATGGAGTG
mQ_f	CAGAGAAGCACTCCATATCCCC
mQ_r	TGGAAAGTAAGCGATTTGGAAGGA
mR_f	CCTTCCAAATCGCTTACTTTCCA
mR_r	GATCTGTTGATGAGTGGCAGGA
mS_f	GCTAAAGGGTTTGTCTACCACTG
mS_r	CTCACACACACACCCTTGTCAC
mT_f	AGGACAAACCAATGACTTCCAC
mT_r	GGCTTGAGAGAAGAGAAGAGTAGAG
mU_f	GAAGAACCAATTTAAATTGATCTA
mU_r	AGAGAACAGGATTCTTCTTGTT
mV_f	CCAAACCAGAACAAGAAGAATCCTG
mV_r	TGGCCGTTCAGTAGATTTAGGAG
mA.1_f	GAGCCCTAAACAAACAGCGC
mA.1_r	CCCAGCAGCTTGTAGAGAGC
mG.1_f	GACATTGGAAGAAGGCTGCG
mG.1_r	TCCTGTAAGAATCCACTCCCAC
mI.1_f	GGAGAGACTAGCAGCTGGAAC
mI.1_r	GACAACCCAAAGCAAGCCG
mK.1_f	TGAGGAGTTCGACCCAAACG
mK.1_r	GGTTTTCTTGTGGTTGGAGGC
mO.1_f	CGGTGGGAATCAAGCGGTAC
mO.1_r	TAAGTCACGTAAACAGCGCG
mQ.1_f	AGCACTCCATATCCCCTTGG
mQ.1_r	GAGAGTCTACACCCTTCCTGG
mV.1_f	TGCAGGGCTGGTTAAGATGG
mV.1_r	CGGGGTTAAGTGTCTCGGTC

Supplementary Table 6: Full-length and mutated sequence of GATA6-AS.

**Full-length GATA6-AS (5'-3')**	**Mutant GATA6-AS (5'-3')**
RefSeq: NR_102763.1	
AACCCCCGCCTAGCCCCTTCACAGCAAATGCGCTTTTTGC<br>CCTGAAGTTGTGTGAGACCGACCTTAAACTTTCTCCGACC<br>CTATCTCGGGATGCTACGGCTCAGGTCGTGGTGGTTTCG<br>GGAACCTCAAGACAACATTCCCCAGCTGACCTTTGGGAA<br>CTTTAACTCGGGTCGTCATGTACGGAAAGGTAGTCTATG<br>GGGTACGCAGAATGGAAAAGGGCTTCCACATCAGTCGT<br>GTCCGAGGACGCTCTCCAACTTTTGATGTCCCTGGAGAG<br>TTTCTGATATTTCCCTGGAGAGTTTCAGAAAGGATTTCTT<br>CCGACAGACGTGACCCCAGAAGGCTGCCGAGGGAGACT<br>TTAGGACAAGAGGCGTTGTTTTTGTGAGAATGAATTTTT<br>ATACTTTACCCCGACCCCACCCCCTACCCCCCGCCCAGGT<br>AAATCCAAGTAAATGATTTTCTGAAGGTCGGTCTCCTAG<br>AAAGCGTTTAGGCTCTGGTTTTTTGTTTTGTTTCTATTTTC<br>GTGTTTTCCCAGTTAGGGAAGGAAGAGGAGCCTGTGCA<br>GAGTTGAACTGGGGTGGAGAGGTGCCTTGTAAAGTATG<br>GTTTCCAGAGATGGTGAGACCTCGTAGCTAAGCGTTGTT<br>TCCTCTGCACGTAACTGTGAGTTGTGAACTTGTGGCTCCT<br>GGGGGAGCGGGACAGGGCCTGGCAGTGCGGGGGAGG<br>GGGCAAGGAATTCAGGAAGTTGGCGAGAATTCTTCGTTT<br>TTCATTCTTCGTTGTAAATTGCTTCTAAACACTTCTGAATT<br>TTAAATTGCTACCGAGAAGGTCCAGAAACCGTTCTCATCC<br>AATTTAGTCACTTTTATTACGTTCCACTTTGCCTCCCCCAA<br>TCACTGCGGCGGCTCCCAGTTTCAGCGTGGCCGCATTTG<br>GAAAAGGCTGAAGGTAGAAAACTCTGGAATGGAGAGGC<br>TGGCGACTGGAGCAGGATCCAGGGGCTTTAAATTTTAAC<br>CTGAATGCGATCGAATGCGCGACTCCCAGAAGAGATAGC<br>CAGGCGTCCGCCCGGCCGCTCAACCCCCGCCAGGACTCG<br>GAATGCCTTGCTTTTGGTTCTTTTCGTGAGTTCCGACCAA<br>ATGTTTAAGTGGACTGGGTTTTGCAGAAGCTCATTTGTA<br>AGCACTGACGGAAATGGGTTGTGGGCATTTGATTTCCTT<br>TGATTCCAACGTTTTGAAATAATGTTTCTCGGATTTGCTA<br>TTCGAAGCAATTTTCTAAAAACTCATATCTTATTTGCTAAA<br>AATAAACTCTTTGGCTCAGTTTGTGTCCAGGCTGTGTACG<br>GCCTCCAACCCTGAGCCCCGAGGAGATCGAGCCCTGGCG<br>CGAGCCTGATCTCTGTCCTGTGTGTTTCTTTGTATTTGCA<br>AGTACAAGGATGTCTGCGTGGATATCCCTGGACACTCGA<br>TACCCCCCGGGGACCTGCTTTTCCCGCAGCTCCGCAGAC<br>GCGGCGGGAGGGCGCGCTGGCCCCGGCGCTGCTGGGA<br>CAGAGGTGAGCAGCGGGGTGGACATTGACCGATTCGAC<br>AACGTTCGGGCTCTAAGGGCTCGGGAAGGGACCCGGCA<br>TTTTACCCTCCAGATTCCCAATCTATTTTTAAAAATAATGT<br>TCAAAACATATGGGAAAAATAAGTGATTGCTCCTTTTCAT<br>GGTCACAGATTGTATTTATTTTTTCAGACTGGTTTTCCAAC<br>CTAACGAGGGAATGGGTAGTGAATGTTGTTGTTGTTGTT<br>GTTGTTTATGTTGGTTAATTTCGAAATAAAACTGTTTAAC<br>TCGTACAGTTGATTCAGACAG	GTGAGTTGTGAACTTGTGGCTCCTGGGGGAGCGGGACA<br>GGGCCTGGCAGTGCGGGGGAGGGGGCAAGGAATTCAG<br>GAAGTTGGCGAGAATTCTTCGTTTTTCATTCTTCGTTGTA<br>AATTGCTTCTAAACACTTCTGAATTTTAAATTGCTACCGA<br>GAAGGTCCAGAAACCGTTCTCATCCAATTTAGTCACTTTT<br>ATTACGTTCCACTTTGCCTCCCCCAATCACTGCGGCGGCT<br>CCCAGTTTCAGCGTGGCCGCATTTGGAAAAGGCTGAAGG<br>TAGAAAACTCTGGAATGGAGAGGCTGGCGACTGGAGCA<br>GGATCCAGGGGCTTTAAATTTTAACCTGAATGCGATCGA<br>ATGCGCGACTCCCAGAAGAGATAGCCAGGCGTCCGCCC<br>GGCCGCTCAACCCCCGCCAGGACTCGGAATGCCTTGCTT<br>TTGGTTCTTTTCGTGAGTTCCGACCAAATGTTTAAGTGGA<br>CTGGGTTTTGCAGAAGCTCATTTGTAAGCACTGACGGAA<br>ATGGGTTGTGGGCATTTGATTTCCTTTGATTCCAACGTTT<br>TGAAATAATGTTTCTCGGATTTGCTATTCGAAGCAATTTT<br>CTAAAAACTCATATCTTATTTGCTAAAAATAAACTCTTTG<br>GCTCAGTTTGTGTCCAGGCTGTGTACGGCCTCCAACCCTG<br>AGCCCCGAGGAGATCGAGCCCTGGCGCGAGCCTGATCT<br>CTGTCCTGTGTGTTTCTTTGTATTTGCAAG
